# Optimizing global health experiences in emergency medicine residency programs: a consensus statement from the Council of Emergency Medicine Residency Directors 2011 Academic Assembly global health specialty track

**DOI:** 10.1186/1865-1380-5-43

**Published:** 2012-11-13

**Authors:** Janis P Tupesis, Christine Babcock, Doug Char, Kumar Alagappan, Braden Hexom, G Bobby Kapur

**Affiliations:** 1 Division of Emergency Medicine, University of Wisconsin School of Medicine and Public Health, 600 Highland Avenue, Madison, WI, 53792, USA; 2 Section of Emergency Medicine, University of Chicago Medical Center, 5841 South Maryland Avenue MC 5068 L-539, Chicago, IL, C60637, USA; 3 Division of Emergency Medicine, Washington University School of Medicine, 660 South Euclid Avenue, Box 8072, St. Louis, MO, 63110, USA; 4 Department of Emergency Medicine, Long Island Jewish Medical Center, 270-05 76th Avenue, New Hyde Park, NY, 11040, USA; 5 Department of Emergency Medicine, Mt. Sinai School of Medicine, One Gustave L. Levy Place, Box 1149, New York, NY, 10029, USA; 6 Section of Emergency Medicine, Baylor College of Medicine, One Baylor Plaza, Suite 134 MS:BCM311, Houston, TX, 77030, USA

**Keywords:** International emergency medicine, Global health, Graduate medical education, Residency training

## Abstract

**Background:**

An increasing number of emergency medicine (EM) residency training programs have residents interested in participating in clinical rotations in other countries. However, the policies that each individual training program applies to this process are different. To our knowledge, little has been done in the standardization of these experiences to help EM residency programs with the evaluation, administration and implementation of a successful global health clinical elective experience. The objective of this project was to assess the current status of EM global health electives at residency training programs and to establish recommendations from educators in EM on the best methodology to implement successful global health electives.

**Methods:**

During the 2011 Council of Emergency Medicine Residency Directors (CORD) Academic Assembly, participants met to address this issue in a mediated discussion session and working group. Session participants examined data previously obtained via the CORD online listserve, discussed best practices in global health applications, evaluations and partnerships, and explored possible solutions to some of the challenges. In addition a survey was sent to CORD members prior to the 2011 Academic Assembly to evaluate the resources and processes for EM residents’ global experiences.

**Results:**

Recommendations included creating a global health working group within the organization, optimizing a clearinghouse of elective opportunities for residents and standardizing elective application materials, site evaluations and resident assessment/feedback methods. The survey showed that 71.4% of respondents have global health partnerships and electives. However, only 36.7% of programs require pre-departure training, and only 20% have formal competency requirements for these global health electives.

**Conclusions:**

A large number of EM training programs have global health experiences available, but these electives and the trainees may benefit from additional institutional support and formalized structure.

## Background

Over the past 20 years, interest in global health opportunities among medical students and residents has been increasing. In 2001, 20% of graduating medical students stated that they had participated in an international/global health training experience during medical school, up from 5% in 1984 [1,2]. In 2011, this number had increased to 30.5% [3]. Experiences from various graduate medical education programs seem to indicate that participating in a global health elective during training exposes trainees to different disease pathologies, teaches them to work in resource limited settings, increases their cultural sensitivity and communication skills, and provides them with an increased awareness of community and public health [4-10]. However, despite the increased interest by resident trainees, little has been done in the standardization of these experiences. Often, residency program directors are charged with identifying the educational merit of global health electives without having first hand knowledge of the experience. Without standardization, large disparities remain in funding, accreditation, oversight and evaluation among EM residency training programs.

## Methods

Each year, CORD convenes program directors, clerkship directors, administrators and other educators at its annual Academic Assembly (AA). As part of the 2011 AA, a specialty track was devoted to evaluate how global health experiences are integrated into EM residency training programs. Two months prior to the conference, all EM residency programs in the US were asked to fill out an online survey (*Surveymonkey,* Palo Alto, CA) querying individual program practices regarding resident global health electives. The survey consisted of nine questions and can be seen in Table 1 below. Survey participants were identified by CORD’s email listserve, representing all residency training programs in the US. A follow-up email was sent through the same listserve 2 weeks prior to the conference.

**Table 1:** Results from global health experiences survey of Council of Emergency Medicine residency directors

**Question**		** Yes**	** No**
1. Do you currently have global health partnerships and global health electives available for residents?		30 (71.4%)	12 (28.6%)
If responded “yes” to question 1: (*n* = 30)			
2a. Does your GME office require a formal application for global health electives?		26 (65%)	4 (35%)
2b. If so, does it require an evaluation of the international training site?		15 (57.7%)	11 (42.3%)
3. Does it require a faculty mentor?		27 (90%)	3 (10%)
4. Does it require a professionalism agreement?		16 (53.3%)	14 (46.7%)
5. Does your GME office fund residents’ stipends during GH electives?		24 (80.0%)	6 (20.0%)
6. Do you have formal pre-departure training for residents that participate in global health electives?		11 (36.7%)	19 (63.3%)
7. Do you provide a post-elective feedback/debriefing session?		20 (66.7%)	10 (33.3%)
8. Do you have a formal competency requirement for residents that elect to participate in global health electives?		6 (20.0%)	24 (80.0%)

During the 2011 AA, approximately 40 residency program directors (PD), associate/assistant program directors (APD), EM faculty members and program coordinators participated in the specialty track: *International Emergency Medicine Electives – Practical Application and Implementation in a Residency Training Curriculum.* The specialty track was divided into three 1-h sessions, each focused on a different aspect of global health in Graduate Medical Education. The focus of the first 2 h was on the “pre-departure” preparatory time frame for both residents and program directors. During this session, the panel discussed regulatory stances by the American Board of Emergency Medicine (ABEM), the Accreditation Council for Graduate Medical Education (ACGME) and the Residency Review Committee (RRC) and the implications of these stances for institutional and individual funding. This was followed by a discussion of the optimization of global health “elective” documentation, both from a programmatic (PD) and institutional (Designated Institutional Official) perspective, using materials supplied from the GME office at the University of Wisconsin. Specific topics included: international site evaluations, emergency plans and procedures, program director/mentor letter of endorsements, pre-departure cultural competency curricula, professionalism agreements, blood-borne pathogen protocols and post-elective resident/site/experience evaluations.

The last part of the specialty track was focused on protocols to optimize EM global health electives for residents. The discussion focused on methods to evaluate these electives prior to the residents’ departure, methods to standardize the evaluative process for residents while on these electives and methods to debrief/evaluate residents upon their arrival back home. One of the main discussion points focused on how to evaluate international training sites for residents in order for the elective to be more effective. It was noted that PDs need a systematic way to evaluate the maturity of EM in a country. Participants noted the challenge of identifying clinical sites that offer sound educational opportunities for the residents − not only ones that are based on geography, safety, logistics and support. At the end of these sessions a working group was convened in order to discuss survey results, give feedback on the session and offer creative solutions to some of the challenges outlined. Members of this working group included Emergency Medicine residency program directors and associate program directors, Emergency Medicine residency program coordinators and academic faculty involved in institutional global health programs. The primary areas of initial focus were the application process for residents to apply for electives, evaluations methods of the electives and areas for partnership.

## Results and discussion

Responses were received from 42 of 155 residency training programs, with a response rate of 27% (Table 1).

The following is a review of the general recommendations from the working group and general sessions:

### Application

1. Global Health Elective Application Standardization: It is recommended that global health elective application materials be standardized. During the consensus proceedings, many of the participants offered to “open source” application materials for other programs to use. It was proposed that the CORD “Sharepoint” website could be used for this in order for the entire organization’s constituency to be able to access these materials.

2. Global Health Elective Application Availability: It is recommended that the global health application materials be made available to individual institutional GME offices, PDs and resident applicants. As above, training programs that have developed “best practices” were encouraged to share their application materials. It is encouraged that these materials be used as they provide a concise, organized and thorough approach for institutional officials to use.

### Evaluation

1. Global Health Site Evaluation: In order to provide the most robust educational experience for the resident trainees and ensure long-term evaluation of site value, it is recommended that a standardized metric or tool be developed to evaluate international training sites. Specifics proposed to be used in this tool included: identifying mentor/supervisor at training site, identifying type of experience (clinical, research, public health), identifying agreed-upon roles and responsibilities for the resident, assuring adequate pre-departure logistics training for the resident (housing, transportation, food, security, immunizations, travel vaccines, travel medication, evacuation insurance, etc.).

2. Resident Evaluation: In order to derive optimal data from these experiences and to provide PDs with essential feedback for monitoring core competency development, it is proposed that a standardized resident clinical evaluation tool be developed. It was proposed that a standardized evaluation of the resident and a standardized resident evaluation of the training site be developed. The resident evaluation was to be a competency-based evaluation, based on the Accreditation Council of Graduate Medical Education’s paradigms of Patient Care, Medical Knowledge, Practice-Based Learning, Interpersonal and Communication Skills, Professionalism and Systems-Based Practice. It was proposed that resident evaluations of the program include: overall educational value of the experience, ease of arranging the elective, level of supervision provided, level of service/support provided, value to career growth and value for personal development.

### Partnership

1. Global Health Site Database: It is recommended to continue to work with other professional organizations (EMRA, SAEM Academy, ACEP, AAEM) and their International Interest groups to provide a comprehensive, real-time clearinghouse of global health opportunities. At the time of the conference, there was an active project by members of the Emergency Medicine Residents Association (EMRA) to collate and publish this data.

2. Global Health Mentoring: In order to foster long-term investment in global health skills development and career mentorship, it is recommended that a virtual mentoring program be developed for residents and faculty at those sites that have limited opportunities.

3. Strategic Planning: It is recommended that a Global Health Education Working Group be developed within CORD to improve and integrate the process by which educational electives are offered and evaluated and promote the standardization with which resident trainees are assessed.

## Discussion

Interest in global health education among medical students, residents, faculty and institutions continues to increase [11-13]. However, the evaluation of a truly “educational” elective and the standardization of applications, funding structures, site and resident evaluations remain elusive. While over 70% of respondent residency programs offer elective opportunities in global health, a third or less have formal competency evaluation for residents prior to participating (20%) and a formalized pre-departure training process (36.7%). Similarly, programs often do not have standardized methods to evaluate the training sites where the residents will be doing their clinical work, and frequently the residents themselves.

A wealth of data, both during medical school and residency training, shows that students and physicians in training find participation in a global health elective to be a valuable experience. They describe enhanced clinical and communication skills, exposure to different disease processes, increased cultural sensitivity, attention to appropriate resource allocation and enhanced community, social and public health awareness. Many of these skill sets are analogous to the ACGME core competencies used to evaluate trainees. However, a paucity of data evaluating resident experiences in these environments persists.

While many other professional organizations in EM have international/global health sections, CORD does not. Given the increasing interest among residents, the poor standardization of processes and the possibility of marked impact on resident education, the working group suggests creating a Global Health Working Group within CORD. In doing so, this resource may decrease the administrative time needed for PDs to offer a robust educational experience, offer guidance to PDs that have limited global health experience/exposure and help navigate the complexities of institutional GME oversight.

## Conclusions

During the 2011 CORD Academic Assembly, a Specialty Track in Global Health training was convened and participants discussed guidelines and best practices for global health training beneficial to the membership of CORD. The Specialty Track developed the following consensus recommendations to enhance the global health education of emergency medicine residents:

Create a Global Health Working Group within CORD

Work with other professional organizations within EM to provide a well-organized, up-to -date clearinghouse of global health opportunities for residents

Standardize global health application materials; make these available to EM program directors

Standardize resident evaluations during global health rotations; make these available to EM program directors

### Limitations

The authors recognize certain limitations to the study. We acknowledge that the response rate to the pre-conference questionnaire and participation in the conference didactic session was limited and may introduce a component of selection bias. We also recognize that some of the recommendations of the consensus conference include participation and partnership with other professional Emergency Medicine organizations.

## Competing interests

The authors declare that they have no competing interests.

## Authors’ contribution

JPT: Study concept and design; acquisition of the data; analysis and interpretation of the data; drafting of the manuscript; critical revision of the manuscript for important intellectual content; statistical expertise; obtained funding; administrative, technical and material support; study supervision. CB, KA, BH: Study concept and design; analysis and interpretation of the data; drafting of the manuscript; critical revision of the manuscript for important intellectual content. DC: Study concept and design; analysis and interpretation of the data; drafting of the manuscript; critical revision of the manuscript for important intellectual content; study supervision. GBK: Study concept and design; administrative, technical and material support; analysis and interpretation of the data; drafting of the manuscript; critical revision of the manuscript for important intellectual content. All authors read and approved the final manuscript.
